# Ameliorating the Adverse Effects of *Tomato mosaic tobamovirus* Infecting Tomato Plants in Egypt by Boosting Immunity in Tomato Plants Using Zinc Oxide Nanoparticles

**DOI:** 10.3390/molecules26051337

**Published:** 2021-03-02

**Authors:** Ahmed R. Sofy, Mahmoud R. Sofy, Ahmed A. Hmed, Rehab A. Dawoud, Abd El-Aleem M. Alnaggar, Ahmed M. Soliman, Noha K. El-Dougdoug

**Affiliations:** 1 Botany and Microbiology Department, Faculty of Science, Al-Azhar University, Nasr City, Cairo 11884, Egypt; ahmed_hmed@azhar.edu.eg; 2 Virus and Phytoplasma Research Department, Plant Pathology Research Institute, Agricultural Research Center (ARC), Giza 12619, Egypt; rehab_dawood2011@yahoo.com (R.A.D.); amohamed@kfu.edu.sa (A.M.S.); 3 Department of Biology, Faculty of Science, Jazan University, Box 114, Jazan 45142, Saudi Arabia; 4 Agriculture Botany Department, Faculty of Agriculture, Al-Azhar University, Nasr City, Cairo 11884, Egypt; abdalaleem_alnaggar.5@azhar.edu.eg; 5 Department of Arid Land Agriculture, College of Agricultural & Food Sciences, King Faisal University, Al-Ahsa 31982, Saudi Arabia; 6 Botany and Microbiology Department, Faculty of Science, Benha University, Benha 13518, Egypt; nohaeldougdoug@gmail.com

**Keywords:** tomato, ToMV, *Tobamovirus*, ZnO-NPs, nanoparticles, boost immunity

## Abstract

*Tomato mosaic virus* (ToMV) is one of the economically damageable *Tobamovirus* infecting the tomato in Egypt that has caused significant losses. It is therefore of great interest to trigger systemic resistance to ToMV. In this endeavor, we aimed to explore the capacity of ZnO-NPs (zinc oxide nanoparticles) to trigger tomato plant resistance against ToMV. Effects of ZnO-NPs on tomato (*Solanum lycopersicum* L.) growth indices and antioxidant defense system activity under ToMV stress were investigated. Noticeably that treatment with ZnO-NPs showed remarkably increased growth indices, photosynthetic attributes, and enzymatic and non-enzymatic antioxidants compared to the challenge control. Interestingly, oxidative damage caused by ToMV was reduced by reducing malondialdehyde, H_2_O_2_, and O_2_ levels. Overall, ZnO-NPs offer a safe and economic antiviral agent against ToMV.

## 1. Introduction

Tomato (*Solanum lycopersicum* L.) is one of Egypt’s most valuable economic vegetable crops. Since tomatoes’ annual production has seen a rising pattern year on year, the occurrence and seriousness of diseases have reduced tomatoes’ yield and quality, resulting in severe losses. Virus-induced diseases are among the most critical factors affecting tomato production in Egypt [1,2].

*Tobamovirus* is the largest genus in the *Virgaviridae* family in which *Tobacco mosaic virus* (TMV) and *Tomato mosaic virus* (ToMV) are two economically damageable *Tobamoviruses* infecting tomato in Egypt [1,2]. ToMV is found in tomatoes worldwide, leading to significant losses [1,3,4].

Many approaches are available for controlling tobamoviruses, including breeding of resistant varieties, biocontrol, and chemical regulation. ToMV is difficult to control where it can be transmitted by seeds and mechanical transmission through grafting or contaminated tools and workers, hence it spreads very quickly [1,3,4]. Therefore, it is urgently needed to advance successful techniques for controlling ToMV infection for preserving agriculture and food production [1,3,4]. Nanotechnology has been studied as an alternative management approach, with nanoparticles (NPs) incorporated into viral plant disease control tools offering important plant safety and health potential [5,6]. The efficacy of NPs can be directly linked to their antiviral function and capacity to activate plant defense mechanisms [5,6]. Since the application of NPs can contribute to an improvement in an enzymatic activity that helps the plant to withstand the virus [6]. Zinc oxide nanoparticles (ZnO-NPs) have also engaged significant attention from a vast number of forms of NPs due to their advantages of being non-toxic, providing strong biocompatibility with human cells, and being simple to acquire [7,8]. ZnO-NPs can be absorbed and transferred across the plant through the leaves, ensuring that synergistic nutritional and immune control can be provided, minimizing the seriousness of the disease [5,6,9].

In continuity to explore new safe antiviral applicants in our outgoing program [6,10,11], the main aims of this study are to investigate the capacity of ZnO-NPs to effectively trigger resistance in tomato plants against the *Tomato mosaic tobamovirus* (ToMV) infection.

## 2. Results and Discussion

### 2.1. Source of the Virus Isolate

**Figure 1:**
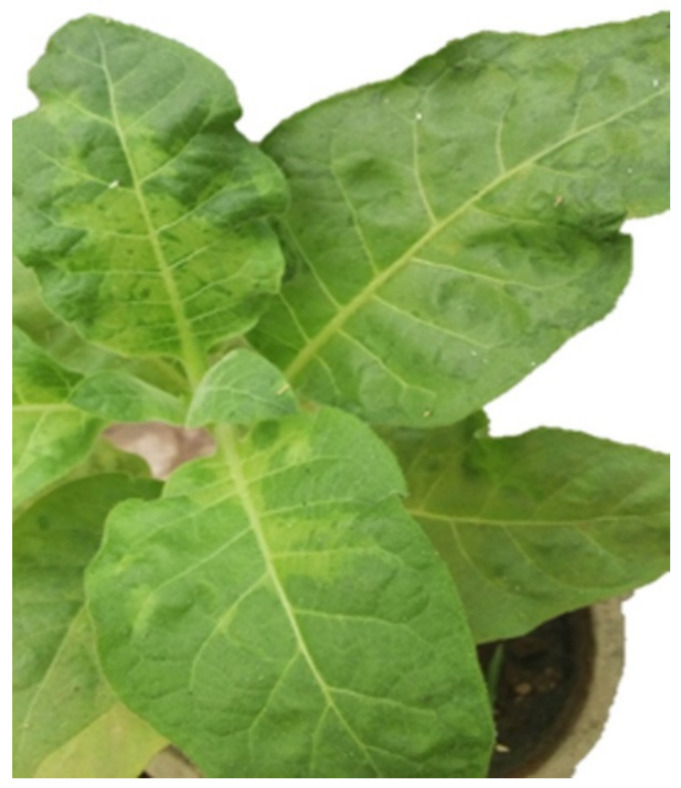
*Nicotiana tabacum* cv. Samsun (B) mechanically inoculated with ToMV-EG, which showed mosaic symptoms.

*Tomato mosaic virus* Egyptian isolate (ToMV-EG) infectious sap has been biologically confirmed by viral infection symptoms in *Nicotiana glutinosa* and *N. tabacum* cv. Samsun. Since it was isolated from homologous local lesions induced on *Nicotiana glutinosa* and then propagated in a healthy *N. tabacum* cv. Samsun as a propagative host, which showed mosaic symptoms (Figure 1). DAS-ELISA testing was conducted to confirm the involvement of the associated virus using the ToMV polyclonal antibody. Our results are consistent with the previous results, which showed similar symptoms [2,12,13].

### 2.2. Improving Negative Effects of ToMV Using ZnO-NPs

#### 2.2.1. Direct Inactivation of ToMV by ZnO-NPs

**Figure 2:**
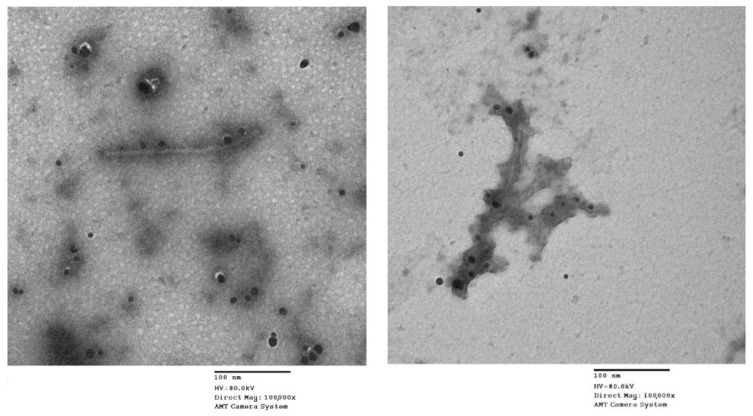
Transmission electron microscope (TEM) images of *Tomato mosaic virus* particles treated with zinc oxide nanoparticles (ZnO-NPs) in vitro for 2 h.

**Figure 3:**
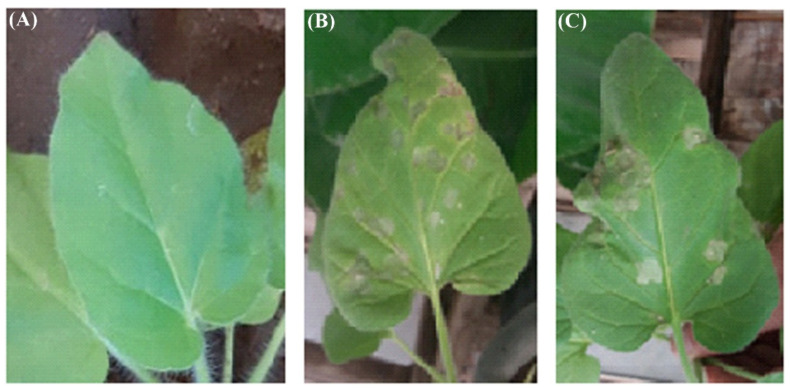
*Nicotiana glutinosa* mechanically inoculated with the two mixtures (*Tomato mosaic virus* particles treated with 50 mg/L of ZnO-NPs (**B**) and 100 mg/L of ZnO-NPs (**C**) in vitro for 2 h compared to healthy (**A**).

It is possible that the direct antiviral effect of ZnO-NPs on ToMV does not suppress viral disease, but the damage and direct disabling of ToMV are of particular importance as ToMV abnormalities demonstrate the primary antiviral function of ZnO-NPs. It, therefore, is proposed first of all that it should be decided whether ZnO-NPs and ToMV interact directly with each other. Transmission electron microscopy (TEM) micrographs of a mixture of ZnO-NPs pre-treated with ToMV for 2 h in vitro showed that the ToMV particles were aggregated and damaged (Figure 2) compared to the untreated ToMV particles, which showed baculiform morphology and distributed equally. Our results agreed with Cai et al. [5], who found that *Tobacco mosaic virus* (TMV) particles were aggregated and damaged after 2 h in vitro treatment with 100 mg/L of ZnO-NPs. As a result, fewer viruses access or multiply in plant cells due to direct damage to the ToMV coat protein or to the burden on the surface of the virus and nanoparticles, or both [5]. This prevents the virus from entering and replicating, which inevitably prevents the virus from multiplying in the host plant and thus inhibits the virus infection in the leaves [5]. Therefore, by mixing 1 mL of ZnO-NPs at different concentrations (50 mg/L or 100 mg/L) with 1 mL of ToMV crude sap, the direct antiviral activity of ZnO-NPs was evaluated by mechanical inoculation of the two mixtures onto *Nicotiana glutinosa* plants. As shown in Figure 3, the rate of ToMV invasion in inoculated leaves was significantly suppressed by the ZnO-NPs at a concentration of 100 mg/L according to the count of local lesions for each concentration. The mean number of local lesions at a concentration of 50 mg/L was significantly higher than that of 100 mg/L, so ZnO-NPs 100 mg/L had higher antiviral activity.

#### 2.2.2. Systemic Protection against ToMV in Tomato Plants Using ZnO-NPs

##### Growth Indices

**Figure 4:**
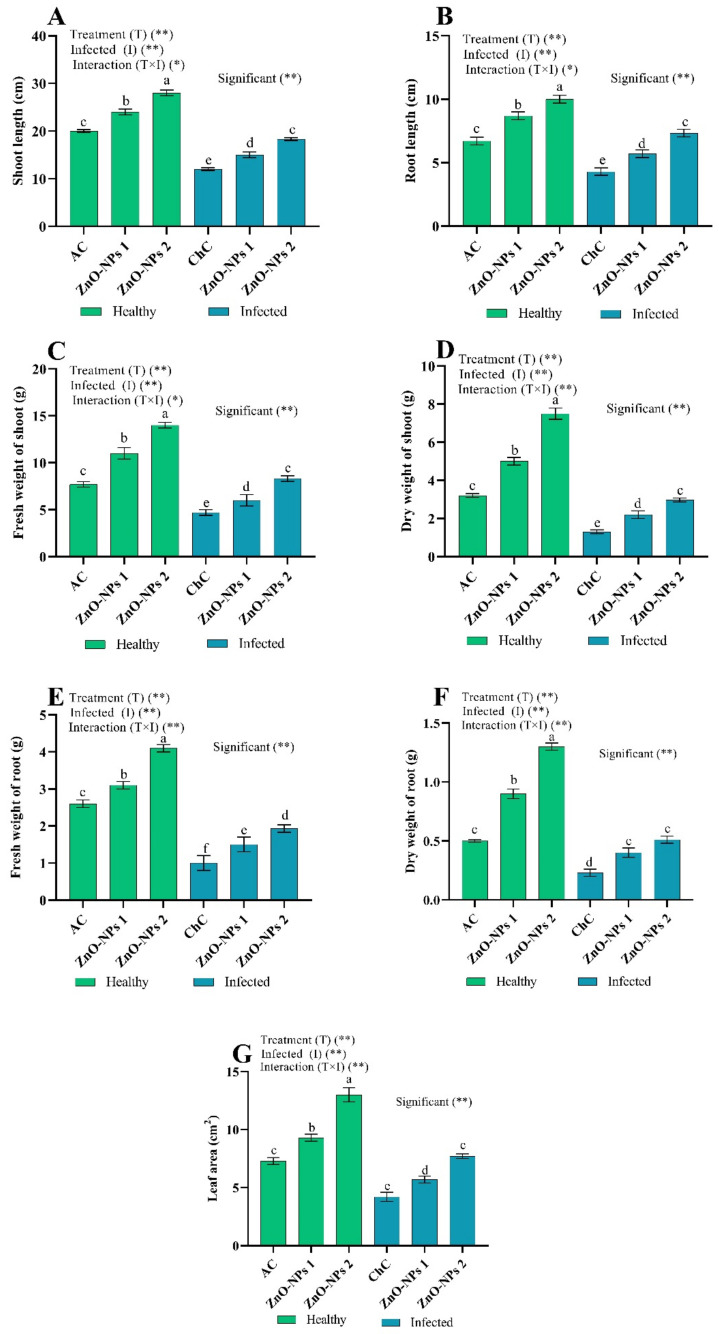
(**A**–**G**) Effect of foliar spray by ZnO-NPs1 (50 mg/L) and ZnO-NPs2 (100 mg/L) on tomato plant growth indices under absolute control (AC) and *Tomato mosaic virus* (ToMV) infection (ChC). According to the Fisher test, the different letters (a, b, c, d, e, f) are significantly different at 0.05%. Vertical bars represent the means of 10 independent determinations ± standard error (SE). * and ** suggest significant and highly significant differences.

The growth indices of 45-day-old tomato plants were measured to show the effect of treatment with the concentrations of 50 mg/L ZnO-NPs (ZnO-NPs1) and 100 mg/L ZnO-NPs (ZnO-NPs2) as a foliar application to non-infected and ToMV-infected plants (Figure 4). ToMV-infected plants (challenge control; ChC) showed a very significant decrease in shoot length and root length (40.00% and 35.82%, respectively), and fresh and dry weight biomass in shoots (38.96% and 61.54%, respectively) compared to the water-treated plants (absolute control; AC) (Figure 4). Similarly, ToMV infection reduced root fresh and dry weight by 61.54% and 54.00%, respectively, and leaf area by 42.47% compared to AC values (Figure 4). However, foliar spraying with ZnO-NPs (ZnO-NPs1 and ZnO-NPs2) significantly alleviated the harmful effects of ToMV infection. Since ZnO-NPs1 increased the shoot and root length by 25.00% and 32.56%, fresh and dry shoot bomass by 27.66% and 46.67%, fresh and dry root biomass by 50.00% and 73.91%, respectively, and leaf area by 35.71%, compared to ChC values. Interestingly, ZnO-NPs2 is more effective than ZnO-NPs1, which significantly increased all growth indices (shoot length, root length, shoot fresh weight, shoot dry weight, root fresh weight, root dry weight, and leaf area) by 52.75%, 70.47%, 77.23%, 98.00%, 93.00%, 121.74%, and 83.33%, respectively, compared to ChC values (Figure 4). Simultaneously, treatments with ZnO-NPs (ZnO-NPs1 and ZnO-NPs2) without inoculation of ToMV did not harm growth indices. At the same time, previous parameters increased compared to corresponding AC values. A similar finding was identified in an earlier study [14]; ZnO-NPs contributed to increased crop production in treated plants and improved the mobilization of native nutrients and soil quality. Additionally, ZnO-NPs are of excellent value because they are cheap to make, safe, and easy to prepare [15], and the US FDA has identified ZnO metal oxide as generally considered safe (GRAS) [16]. Accordingly, our study shows that ZnO-NPs2 could guard plants and increase agricultural production. These results can be appraised to ensure that ZnO-NPs can control plant virus-related diseases as environmentally friendly agents. NPs caused different morpho-physiological variations based on their chemical composition, size, surface contact, and, in particular, dose [17,18]. The most prevalent impact of virus stress on plant physiology is the growth deficiency that is critical to the survival of a plant that has been exposed to this stress [6,19,20,21,22,23,24]. The current study showed that ToMV could induce serious disease, contributing to a decline in growth indices that the exogenous use of ZnO-NPs could ameliorate. ZnO-NPs are considered useful components for plant growth, where the amount of Zn increased in plants [25,26]. Since Zn is an essential micronutrient in many physiological and metabolic processes, it is required for tryptophan synthesis, a precursor to IAA [27]. In addition, Zn plays a crucial role in preserving the integrity of the cell membrane [28]. It is often used to produce proteins, elongation of cells, membrane function, and environmental stress resistance [29,30]. Furthermore, Faizan et al. [31] revealed that ZnO-NPs treatment of the tomato plant resulted in a substantial increase in growth biomarkers. ZnO-NPs treatment also shows a significant improvement in plant biomass in *Solanum lycopersicum* [17]. 

##### Chlorophyll Content and Photosynthetic Characteristics

**Figure 5:**
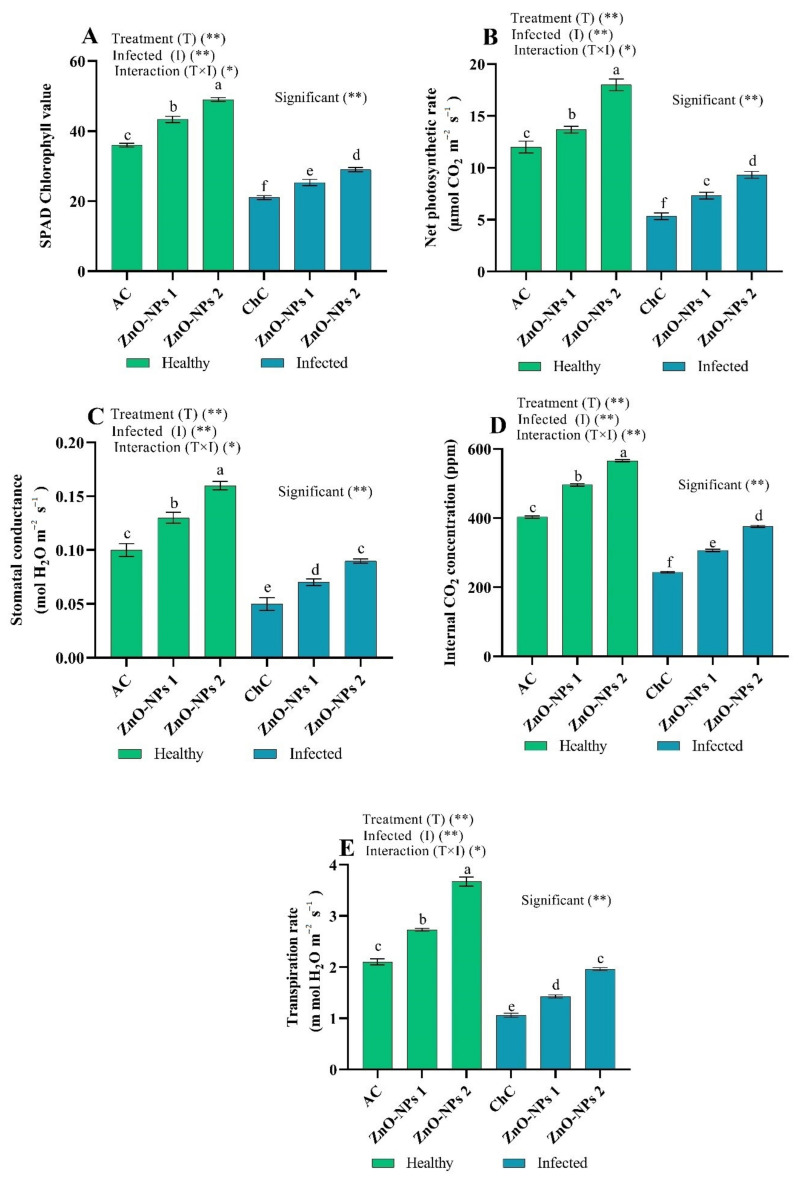
(**A**–**E**) Effect of foliar spray by ZnO-NPs1 (50 mg/L) and ZnO-NPs2 (100 mg/L) on tomato chlorophyll content and photosynthetic characteristics under absolute control (AC) and *Tomato mosaic virus* (ToMV) infection (ChC). According to the Fisher test, the different letters (a, b, c, d, e, f) are significantly different at 0.05%. Vertical bars represent the means of three independent determinations ± standard error (SE). * and ** suggest significant and highly significant differences.

As shown in Figure 5, SPAD chlorophyll values and the photosynthetic characteristics (net photosynthetic rate (PN), stomatal conductance (gs), intercellular CO_2_ concentration (Ci), and transpiration rate (E)) decreased by 41.67%, 55.58%, 50.00%, 39.53%, and 49.52%, respectively, in leaves with higher levels of ToMV symptoms in the ChC sample compared to AC values. On the other hand, the values of SPAD chlorophyll and the photosynthetic characteristics increased in the plant by foliar application of ZnO-NPs (ZnO-NPs1 and ZnO-NPs2). Since the adverse effects of ToMV were mitigated by two concentrations, where the values of SPAD chlorophyll increased by 20.62% and 38.10%, respectively, PN increased by 37.52% and 75.05%, respectively, gs increased by 40.00% and 80.00%, respectively, Ci increased by 25.85% and 54.29%, respectively, and E increased by 34.91% and 84.91%, respectively, as compared to ChC values (Figure 5). In addition, the two ZnO-NPs treatments of virus-free plants presented an improvement in SPAD chlorophyll values and photosynthetic characteristics compared to AC values (Figure 5).

Related findings found that viral infection triggered a decline in photosynthetic pigments of the tomato plant [32] and cucumber plant [33,34]. In virus-infected plants, chlorophyll content reduction can be induced by stimulating various cellular enzymes such as chlorophyllase [35] or the virus’s effect on pigment synthesis [36]. Tomato plants treated with various ZnO-NPs concentrations displayed an increase in photosynthetic pigments, suggesting that the virus may be destroyed by ZnO-NPs, thereby enhancing the host’s tolerance to disease [37]. Govorov and Carmeli [38] stated that NPs could trigger chemical energy efficiency in photosynthetic systems. In addition, Noji et al. [39] stated that photosynthesis II (PSII)-bound nano-sized metal compounds induced stable photosynthetic oxygen-evolving reaction behavior, suggesting the transport of light-driven electrons from water to molecules of quinone, and indicated that PSII conjugate might have photosensor and artificial photosynthetic device properties for growth. NPs increase the photosynthetic rate by enhancing CA and photosynthetic pigment synthesis [40,41]. These modified processes’ cumulative impact could improve photosynthetic machinery in plants exposed to ZnO-NPs2 in a non-stressed or stressed state. These results are compatible with the earlier findings of Faizan et al. [31], which showed that ZnO-NPs raised the Chl content of tomato leaves.

##### Oxidative Stress Markers

**Figure 6:**
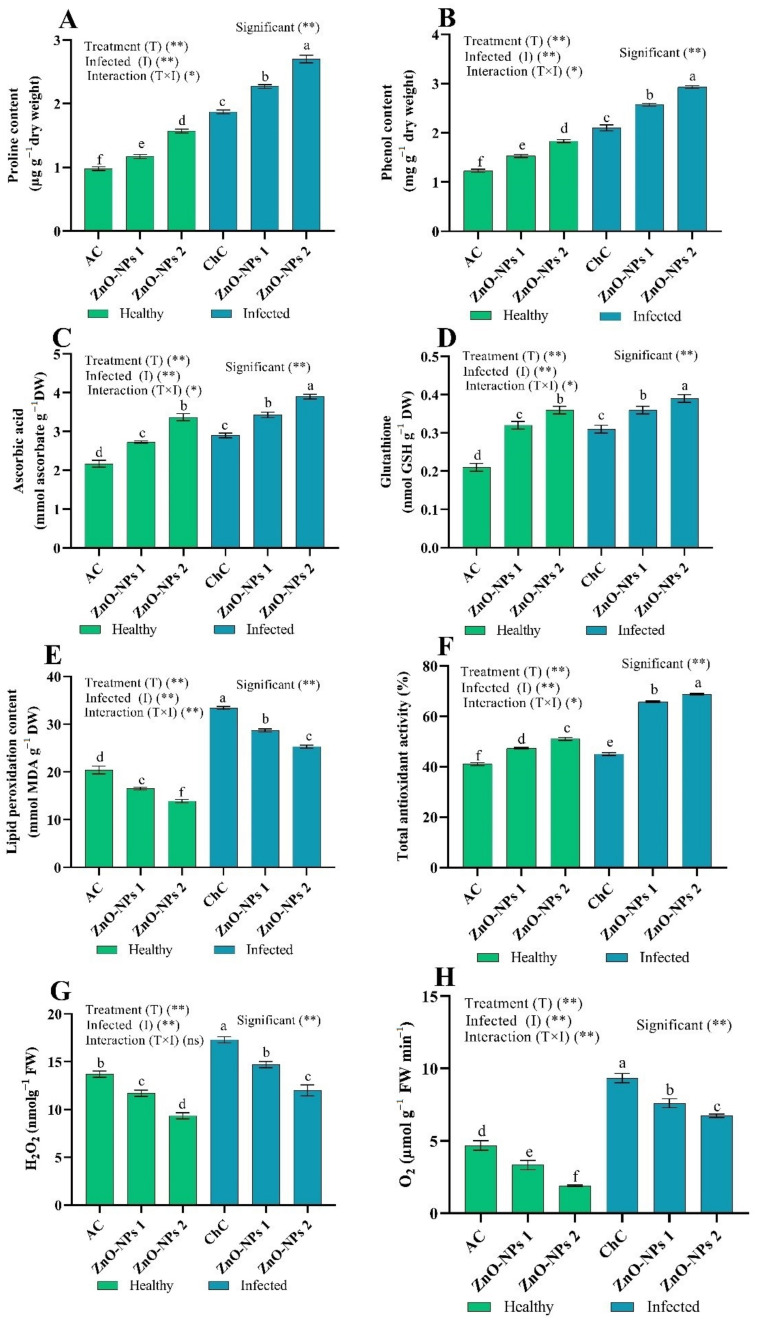
(**A**–**H**) Effect of foliar spray by ZnO-NPs1 (50 mg/L) and ZnO-NPs2 (100 mg/L) on tomato oxidative stress markers under absolute control (AC) and *Tomato mosaic virus* (ToMV) infection (ChC). According to the Fisher test, the different letters (a, b, c, d, e, f) are significantly different at 0.05%. Vertical bars represent the means of three independent determinations ± standard error (SE). * and ** suggest significant and highly significant differences. ns = not significant.

ToMV-infected tomato leaves (ChC) showed a significant increase in the contents of oxidative stress markers (proline, phenol, ascorbic acid (AsA), glutathione (GSH), lipid peroxidation (MDA), total antioxidant activity (TAA), hydrogen peroxide (H_2_O_2_), and oxygen (O_2_) content) by 90.82%, 70.73%, 33.64%, 47.62%, 63.73%, 9.76%, 26.28%, and 99.79%, respectively compared to AC values. Interestingly, ToMV-infected tomato leaves pre-treated with ZnO-NPs (ZnO-NPs1 and ZnO-NPs2) showed a significant increase in proline by 21.39% and 44.39%, respectively, phenol by 22.38% and 39.52%, respectively, AsA by 18.28% and 34.48%, respectively, GSH by 16.13% and 25.81%, respectively, and TAA by 46.00% and 52.67%, respectively compared to ChC values. While MDA decreased by 14.07% and 24.25%, respectively, H_2_O_2_ decreased by 15.03% and 30.64%, respectively, and O_2_ decreased by 18.54% and 27.87%, respectively, compared to ChC values (Figure 6). Metal-based NPs have caused unregulated development of reactive oxygen species (ROS) at multiple plant sites in plants subjected to some external conditions, such as environmental influences, biotic stress (viral, fungi, and bacteria), etc. [6,25,42]. The greater ROS levels trigger oxidative damage, containing lipid, pigment destruction, proline, phenol, ASA, and GSH, and ROS impairs enzyme activities [43]. So that organelles have developed antioxidant defence systems to protect plant cells from oxidative damage by scavenging ROS [44,45,46,47]. Proline is a non-enzymatic antioxidant capable of stabilizing subcellular materials such as cell membranes, proteins, buffering redox potential, and scavenging free radicals under stress conditions. In addition, it has the capacity of molecular chaperones to maintain protein validity and improve the functioning of various enzymes, such as nitrate reductase resistance under biotic stress conditions [48,49]. Proline is also the only compatible molecule to protect plants against single oxygen and radical damage caused by excess ROS, among the many compatible solutes [45]. This study confirms the present findings, in which the treatment of ZnO-NPs improved proline aggregation (Figure 6A). Supplemented ZnO-NPs in Murashige and Skoog medium promoted synthesis of proline and activity of catalase (CAT), superoxide dismutase (SOD), and peroxidase (POX) as well as increased biotic stress tolerance in bananas [50].

##### ROS Scavenging Enzymes

**Figure 7:**
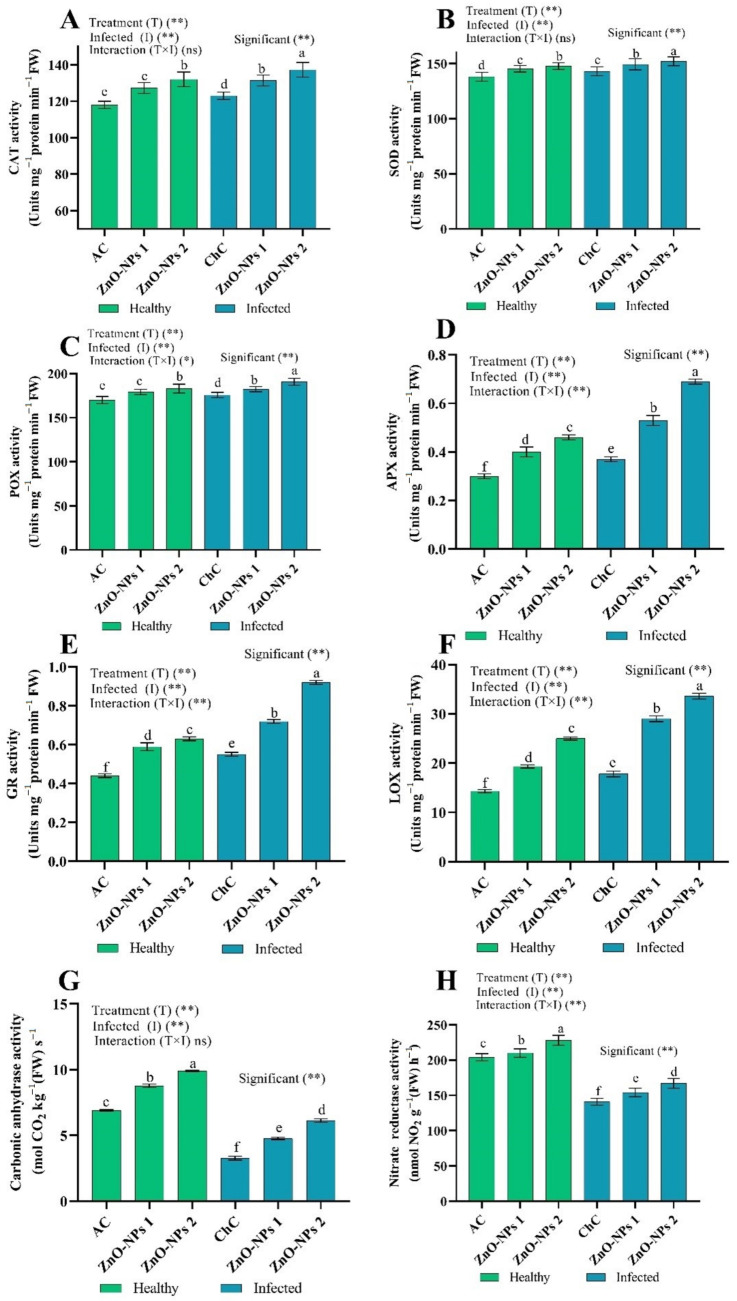
(**A**–**H**) Effect of foliar spray by ZnO-NPs1 (50 mg/L) and ZnO-NPs (100 mg/L) on ROS scavenging enzymes under absolute control (AC) and *Tomato mosaic virus* (ToMV) infection (ChC). According to the Fisher test, the different letters (a, b, c, d, e, f) are significantly different at 0.05%. Vertical bars represent the means of three independent determinations ± standard error (SE). * and ** suggest significant and highly significant differences. ns = not significant.

The pathological effects of ToMV compared to the corresponding AC values resulted in an increase of 4.24%, 3.62%, 3.39%, 23.33%, 25.00%, and 24.48% in catalase (CAT), superoxide dismutase (SOD), peroxidase (POX), ascorbate peroxidase (APX), glutathione reductase (GR), and lipoxygenase (LOX) activity, respectively, with a decrease of 52.61% and 30.88% in carbonic anhydrase (CA) and nitrate reductase (NR), respectively. Interestingly, the foliar application of ZnO-NPs (ZnO-NPs1 and ZnO-NPs2) induced an antioxidant defense system in tomato plant leaves with or without ToMV stress than ChC and AC controls. Under ToMV stress, ZnO-NPs1 and ZnO-NPs2-pretreated tomato plants presented significant rises in CAT by 6.88% and 11.65%, respectively, SOD by 4.43% and 6.29%, respectively, POX by 3.75% and 8.57%, respectively, APX by 43.24% and 86.49%, respectively, GR by 30.91% and 67.27%, respectively, LOX by 62.92% and 88.76%, respectively, CA by 45.87% and 87.46%, respectively, and NR by 9.22% and 18.44%, respectively compared to ChC values (Figure 7).

Plants have a repertoire of mechanisms to counteract and overcome viral stress. One such mechanism, which plays a key role in stabilizing and avoiding oxidative damage, is the non-enzymatic and enzymatic defense antioxidant system. RNS and/or ROS -quenching enzymes such as CAT, SOD, POX, APX, GR, LOX, CA, and NR are the critical elements of the antioxidant defense system [51,52]. Different biotic stresses accelerate the generation of ROS, including O_2_, 1O_2_, OH, and H_2_O_2_, resulting in oxidative stress [53,54] and/or RNS, the major RNS comprises NO and NO_2_ radicals, along with non-radicals, including N_2_O_4_, HNO_2_, NO-, and ONOO- [55,56]. Cellular organelles components, such as nucleic acids, proteins, and lipids, are impaired by oxidative stress, which in turn interferes with regular membrane functions and cell metabolism, leading to lipid peroxidation and eventually programmed cell death [57]. Therefore, regulation of ROS and/or RNS output is necessary to avoid injurious ROS and/or RNS impacts and ensure that their signaling functions are appropriately implemented [58]. Many defense mechanisms have been established by plants to organize both the development and removal of ROS and/or RNS to escape oxidative harm and signal activity [44,59]. The antioxidant protection mechanism effectively eliminates excess ROS by controlling the activity of various enzymes, including CAT, SOD, APX, and GR, in addition to several non-enzymatic reactions [51,60]. SOD is an important enzymatic antioxidant for all aerobic species susceptible to oxidative stress induced by ROS. CAT is an enzyme that can dismute H_2_O_2_ directly into H_2_O and O_2_ and is necessary for the detoxification of ROS under adverse conditions [52]. Peroxidase plays a crucial role in defending higher plants’ cells by scavenging H_2_O_2_ in water-water and glutathione-ascorbate cycles [44]. ToMV decreases the activity of LOX, which induces enhanced lipid peroxidation. Interestingly, oxidative stress in tomato plants was reduced during the recovery time, as confirmed by the decrease in ROS and H_2_O_2_, O_2_ levels and the decrease in MDA content by ZnO-NPs therapy, in line with the outcomes recorded by Lv et al. [61] and Mathioudakis et al. [62]. However, this cycle is an essential component, namely AsA and APX, in addition to scavenging toxic hydrogen peroxide and converting it to H_2_O [63]. In addition, treatment with ZnO-NPs significantly increased antioxidant enzymes in this study (CAT, SOD, POX, APX, GR, LOX, CA, NR; Figure 7). It has also been reported that metal-NPs increase the seed germination in tomato and antioxidant systems under stress conditions [40]. Sofy et al. [6] found that the treatment of NPs showed an increase in growth biomarkers in faba bean plants with an increase in biochemical characteristics (oxidative stress markers and antioxidant enzymes).

##### Correlation Analysis

**Figure 8:**
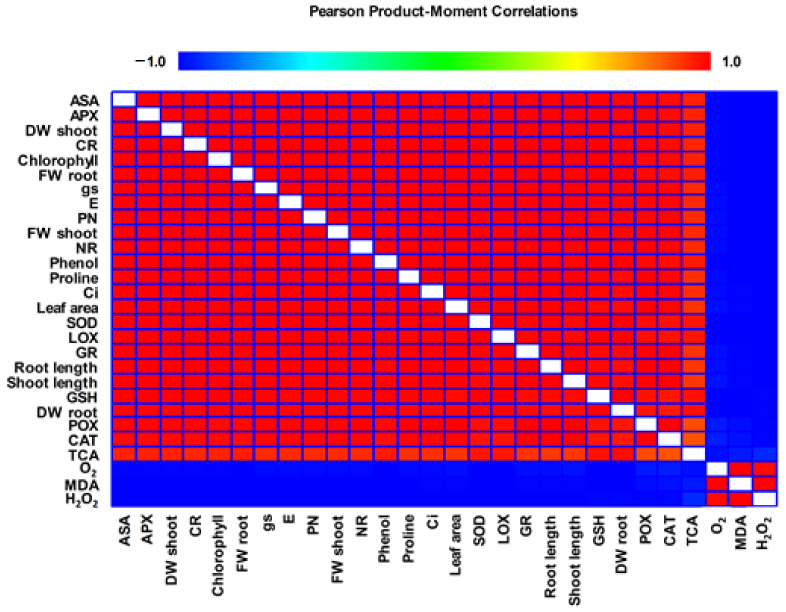
Heat map showing the correlation analysis under ToMV and treatments. The color scale displays the intensity of normalized mean values of different parameters. −0.01 indicate to negative correlation, 0.01 indicate to positive correlation.

The result of the correlation analysis under ToMV and treatments showed that chlorophyll content (SPAD values), photosynthetic characteristics (stomatal conductance (gs), transpiration rate (E), net photosynthetic rate (PN), and intercellular CO_2_ concentration (Ci)), growth indices, ROS scavenging enzymes (peroxidase (POX), catalase (CAT), superoxide dismutase (SOD), lipoxygenase (LOX), nitrate reductase (NR), carbonic anhydrase (CR), and ascorbate peroxidase (APX)), glutathione (GSH), total antioxidant activity (TCA), lipid Peroxidation (MDA), proline, phenol, H_2_O_2_, and O_2_ parameters had significant correlation (Figure 8). There was a positive significant correlation among the ROS scavenging enzymes and chlorophyll content, photosynthetic characteristics, growth indices. In contrast, there was a negative significant correlation between the ROS scavenging enzymes and H_2_O_2_, MDA, O_2_.

## 3. Materials and Methods

### 3.1. Source of the Virus Isolate

*Tomato mosaic virus* (ToMV) Egyptian isolate (ToMV-EG) was maintained on *Solanum lycopersicum*. The virus isolate was confirmed by grinding infected leaf samples in a phosphate buffer (100 mM, pH 7.2) containing 0.2% mercaptoethanol and then mechanically inoculating infectious sap on *Nicotiana glutinosa* and *N. tabacum* cv. Samsun. Since it was isolated from homologous local lesions induced on *N. glutinosa* and then propagated in a healthy *N. tabacum* cv. Samsun as a propagative host. Inoculated plants were kept in an insect-proof greenhouse and inspected daily for symptom expression. The same number of healthy seedlings and age inoculated with buffer only is used as control. The results were confirmed by a double-antibody sandwich enzyme-linked immunosorbent assay (DAS-ELISA) using ToMV polyclonal antibody according to Clark and Adams [64].

### 3.2. Zinc Nanoparticles

The required concentration of ZnO-NPs was prepared by dissolving 50 or 100 mg ZnO-NPs in 1000 mL of double-distilled water (DDW). Four drops of 80% Tween were used with every prepared solution to maximize dissemination on tomato leaves.

### 3.3. Evaluation of Direct Antiviral Action

ToMV was purified from *N. tabacum* cv. Samsun leaves that were systemically infected, according to Gooding and Hebert [65] and Abdelmoamen et al. [12].

In vitro, purified ToMV particles were mixed with tested antiviral materials (ZnO-NPs 50 mg/L or ZnO-NPs 100 mg/L), and the mixtures remained in vitro for 2 h at 25 °C [5]. Subsequently, 10 μL of each mixture was applied to carbon-coated grids and negatively stained with 2% uranyl acetate, pH 7.0, according to Christie et al. [66]. ToMV particle morphology was examined by transmission electron microscopy operated at 80 KV at the Regional Center for Mycology and Biotechnology, Al-Azhar University, Cairo, Egypt.

In vivo, mixtures at 2 h of longevity were rubbed onto carborundum-dusty *Nicotiana glutinosa* leaves to be inoculated with ToMV. The suppression activity (virucidal) of the two concentrations of ZnO-NPs against ToMV infection was assessed by surveying the rate of ToMV invasion of inoculated leaves based on the count of local lesions.

### 3.4. Systemic Protection against ToMV in Tomato Plants Using ZnO-NPs

#### 3.4.1. Plant Materials

The tomato seeds (*Solanum lycopersicum* L.) have been obtained from the ARC, Giza, Egypt. The seeds of good appearance and uniform size were surface-sterilized for 10 min with 1% sodium hypochlorite solution, accompanied by continuous washing with double-distilled water (DDW). The sterilized seeds were planted in a plastic cup to create a nursery. After seven days of sowing, the seedlings were subsequently transplanted into the well-maintained 40 cm pots comprising a sterile soil mixture of 35% clay, 35% sand, and 30% peatmoss, with the temperatures in the day at 27 °C and at night at 23 °C. The plants have been divided into six groups after 14 days of growth. Each group consists of five replicate pots (three healthy plants/pot). The groups were divided into:

1. In the first group, the plants were sprayed with water as untreated control (absolute control) (AC).
2. In the second group, the plants were foliar sprayed with 50 mg/L ZnO-NPs (ZnO-NPs1).
3. In the third group, the plants were foliar sprayed with 100 mg/L ZnO-NPs (ZnO-NPs2).
4. In the fourth group, the plants were inoculated with *Tomato mosaic virus* (ToMV) as challenge control (ChC).
5. In the fifth group, the plants were foliar sprayed with 50 mg/L ZnO-NPs and then inoculated after three days with ToMV (ZnO-NPs1 + V).
6. In the sixth group, the plants were foliar sprayed with 100 mg/L ZnO-NPs and then inoculated after three days with ToMV (ZnO-NPs2 + V).

The control and treated plant samples were taken 21 days after inoculation for analysis.

#### 3.4.2. Growth Indices

The plants were taken out of the pots and put in a water-filled bucket. They were moved lightly to eliminate the agglutinated soil particles, then a meter scale was used to measure the lengths of the shoot and root. The leaf area was determined by a graph sheet on which leaf squares were counted to represent leaf area.

#### 3.4.3. Chlorophyll Content and Photosynthetic Characteristics

A SPAD-502 chlorophyll meter (Konica, Minolta, Inc., Tokyo Japan) was applied to measure chlorophyll SPAD values in tomato leaves [31]. The photosynthetic characteristics (stomatal conductance (gs), transpiration rate (E), net photosynthetic rate (PN), and intercellular CO_2_ concentration (Ci)) in the completely extended uppermost plant leaves were determined by the portable photosynthetic technique in each treatment (LI-COR 6400, LICOR, Lincoln, NE, USA). Relative humidity, photosynthetic photon flux density, CO_2_ concentration, and air temperature were maintained at 85%, 800 μmol mol^−2^ s^−1^, 600 ppm, and 25 °C [31].

#### 3.4.4. Oxidative Stress Markers

##### Proline and Phenols

The proline content of dry tomato leaves was calculated using the Bates et al. [67] method. The material was extracted in sulfosalicylic acid, and the equivalent amount of glacial acetic acid and ninhydrin solution. The sample was heated to 100 °C and after cooling, 5 mL of toluene was applied. On a spectrophotometer, the absorbance of the toluene layer was read at 528 nm.

According to Galicia et al. [68], phenols were tested by the Folin–Ciocalteu reagent (FCR) and Na_2_CO_3_ solution. A total of 6.5 mL of methanol (50%) was combined with one hundred milligrams of dry tomato leaves. The samples were vortexed, permitted to stand at darkroom temperature for 95 min, and then centrifuged for 5 min at 15,000× *g*. Five milliliters of phosphoric acid (85%), 0.8 mL of 25% FCR, and 10 mL of distilled HCl were added to one milliliter of the supernatant. At 42 °C, the tubes were then incubated for 10 min. Then, using a spectrophotometer, the absorption was calculated at 765 nm.

##### Ascorbic Acid (ASA) and Glutathione (GSH) Content

Five-hundred grams of dried tomato leaves were extracted from 6% (*w/v*) trichloroacetic acid (TCA). Two milliliters of dinitro-phenylhydrazine (2%) was then mixed with the extract, accompanied by a reduction of thiourea (10%) dissolved in ethanol (70%). The mixture was boiled for 15 min. After cooling the samples at room temperature, 5 mL of 80% H_2_SO_4_ was added at 0 °C. The absorbance was calculated at 530 nm using a spectrophotometer to calculate the ascorbic acid (ASA) material, as defined in the Mukherjee and Choudhuri [69] method.

In tubes containing 5 mL of metaphosphoric acid (2%), 50 mg of dried tomato leaves were extracted, followed by centrifugation for 10 min at 17,000× *g*. With 0.8 mL of sodium citrate 10%, the supernatant (1 mL) was mixed. A blend of 700 μL NADPH (0.3 mM), 100 μL 5.5′-dithio-bis-2-nitrobenzoic acid (6 mM), 100 μL DDW, and 100 μL extract was stabilized at 25 °C for 4–5 min. GSH reductase (15 μL of 60 units mL^−1^) was supplemented into the extract. A spectrophotometer was used to read absorbance at 412 nm to determine the GSH content by the Griffith [70] method.

##### Lipid Peroxidation and Total Antioxidant Activity

Lipid peroxidation was assessed and quantified concerning malondialdehyde (MDA), as explained by Heath and Packer [71], lipid peroxidation was assessed and quantified with respect to MDA. Half a gram of the tomato leaves was homogenized in 3 mL of 0.2% TCA, accompanied by 25 min of centrifugation at 15,000× *g*. The supernatant (1 mL) obtained was mixed with an equivalent volume of TCA (10%) composed of TBARS (0.5%), heated at 95 °C for 30 min, then cooled, and then checked for supernatant absorption at 532 and 600 nm.

The leaf extracts free radical-scavenging operation was calculated as defined by Brand-Williams et al. [72]. Every methanol extract was combined with 2 mL of a freshly prepared methanol solution having 1,1-diphenyl-2-picrylhydrazyl radicals (80 mg L^−1^). The mixture was vigorously shaken, carried for 30 min in the dark, during which the absorption was read at 517 nm. The DPPH percentage operation was computed using the following Equation (1):

(1) $$\mathrm{DPPH}\;\mathrm{scavenging}\;\mathrm{ability}=\left[1-\left(A_{i}-A_{j}\right)∕A_{c}\right]\times 100$$

where A_i_ is the absorbance of DPPH + extract, A_j_ is the absorbance of methanol + extract, and Ac is the absorbance of methanol + DPPH.

##### Quantification of H_2_O_2_ and O_2_

According to a previous protocol with improvements [73], an analysis of H_2_O_2_ was carried out. Briefly, in an ice bath, 0.5 g leaf samples were homogenized with 5% TCA. At 12,000× *g*, the homogenate was centrifuged, and the supernatant was blended with TiCl_2_. In H_2_SO_4_, the residue was obtained and resuspended. The absorbance of the solution was calculated at 410 nm.

The production of O_2_ was determined as mentioned above [74]. In the phosphate buffer (pH 7.8), leaf samples were homogenized and then centrifuged for 10 min at 12,000× *g*. Then, the supernatant was combined with hydroxylamine hydrochloride. The mixture was then incubated for 1 h at 25 °C and was then blended for 20 min with sulfanilamide and alpha-naphthylamine at 25 °C. Absorption was estimated at 530 nm.

#### 3.4.5. ROS Scavenging Enzymes

In this respect, 3 g of fresh tomato leaves were combined with 15 mL of a phosphate buffer and centrifuged at 2 °C for 20 min at 15,000× *g*. The activity of catalase (CAT) was assessed by the Chen et al. [75] method. The reaction mixture was added to a final amount of 10 mL comprising 9.96 mL of H_2_O_2_ phosphate buffer (0.1 M, pH 6.8). With a UV spectrophotometer at 250 nm, the rate shift of H_2_O_2_ absorbance in 60 s. Superoxide dismutase (SOD) was calculated by pyrogallol autooxidation inhibition by Marklund and Marklund [76]. Next, 0.1 mL of enzyme, 3.6 mL of distilled water, 5.5 mL of phosphate buffer, and 0.8 mL of 3 mM pyrogallol was mixed with the solution. The pyrogallol reduction rate was calculated at 325 nm using a UV-spectrophotometer. A solution comprising 0.2 mL of enzyme extract, 5.8 mL of phosphate buffer, and 2 mL of 20 mM H_2_O_2_ was used to evaluate peroxidase (POX). After the addition of 3 mL of 20 mM pyrogallol, the increase in pyrogallol absorption was measured within 60 s at 470 nm and 25 °C using the UV spectrophotometer [77]. Ascorbate peroxidase (APX) activity was assayed by the method of Nakano and Asada [78]. The reaction mixture contained potassium phosphate buffer, five mM ascorbate, 0.5-mM H_2_O_2_, and enzyme extract. The absorbance was read at 265 nm. According to Jiang and Zhang [79], glutathione reductase activity (GR) was assessed following NADPH oxidation at 340 nm for 1 min. Lipoxygenase activity (LOX) was assessed, where the absorbance was read at 234 nm as described by Todd et al. [80].

Nitrate reductase (NR) activity was assessed using the Jaworski [81] method. Fresh leaf samples were moved to plastic vials containing KNO3, phosphate buffer, and isopropanol and were incubated at 30 °C for 2 h. Sulfanilamide and N-1-naphthylethylenediaminehydrochloride solutions were added after incubation, and the absorbance was read at 540 nm on a UV spectrophotometer. Carbonic anhydrase (CA) activity in the leaves was determined on the basis of Dwivedi and Randhawa [82], where the leaves were blotted and moved to the test tube, together with the addition of 0.4 M NaHCO_3_, phosphate buffer, bromothymol blue, and finally the addition of the methyl red indicator. Enzyme activity was expressed on a fresh weight basis.

#### 3.4.6. Statistical Analysis

The study planning was completely randomized, so all statistical tests were performed at a probability level of 0.05 by SPSS (Statistical Package for the Social Science Version 26.0) [83]. The quantitative findings were calculated using a two-way ANOVA for a Fisher’s test variance study for a Levene test’s parametric distribution. The confidence interval was determined at 95%. The heat map correlation was done between parameters at a confidence interval of 5%.

## 4. Conclusions

Plants can typically tolerate disease infection by proline production, phenol, ascorbic acid, glutathione, total antioxidant activity, and increased antioxidant enzymes. The results showed that foliar spray with ZnO-NPs can mitigate the adverse effect caused by ToMV infection. The ZnO-NPs application induced systemic acquired resistance by scavengers of ROS, accumulated phenolic compounds, ascorbic acid content, and proline production. In conclusion, spraying ToMV-infected tomato plants with ZnO-NPs2 (100 mg/L ZnO-NPs) may be a hopeful strategy to overcome the ToMV infections by triggering the antioxidant defense system.

## Data Availability

Not Applicable.

## Sample Availability

Samples of the compounds are not available from the authors.
